# Iodine content of frequently used prenatal and adult multivitamins in Switzerland

**DOI:** 10.3389/fendo.2022.1041232

**Published:** 2022-11-03

**Authors:** Mandy Gfeller, Gentiane Colque, Peter A. Kopp

**Affiliations:** ^1^ Division of Endocrinology, Diabetes and Metabolism, University Hospital of Lausanne and University of Lausanne, Lausanne, Switzerland; ^2^ Endocrinology, Diabetes and Nutrition, Médical Center EndoDia-Centre, Biel-Bienne, Switzerland; ^3^ Division of Endocrinology, Metabolism and Molecular Medicine, Feinberg School of Medicine, Northwestern University, Chicago, IL, United States

**Keywords:** Iodine, iodine deficiency, pregnancy, thyroid function, multivitamins

## Abstract

**Background:**

Iodine is essential for the biosynthesis of thyroid hormones, which are crucial for intrauterine growth and fetal neurocognitive development. Iodine requirements increase during pregnancy and lactation. The World Health Organization and the Swiss Confederation recommend a total daily iodine intake of 250 µg of iodine during preconception, pregnancy and lactation. To assure this goal, several professional organizations recommend complementing the nutritional iodine intake with supplements containing 150 μg of iodine daily.

**Methods:**

Prenatal and adult multivitamins widely available in Switzerland were compiled to determine their iodine content. Obstetricians verified that the list includes the most frequently prescribed supplements in Switzerland.

**Results:**

A total of 44 adult multivitamin supplements were identified, 23 of which are specifically intended for women planning pregnancy, pregnant, or breastfeeding. Seven out of 23 (30.4%) prenatal multivitamins products, and 12/21 (57.1%) adult multivitamins contained no iodine. Among all the products, only 18/44 (40.9%) contain 150 µg of iodine or more.

**Conclusion:**

Several widely used products contain no or insufficient amounts (<150 ug) of iodine. Providers need to be informed about the variability in iodine content of supplements and established recommendations, and manufacturers of prenatal supplements should assure that their products contain iodine in adequate amounts.

## Introduction

Iodine is an essential component for the production of the thyroid hormones thyroxine (T4) and triiodothyronine (T3). Normal levels of thyroid hormones are crucial for intrauterine growth and neurocognitive development of the fetus, and they are dependent on sufficient maternal dietary iodine intake. The consequences of iodine deficiency (ID) during fetal development and infancy are well established and can range from subtle changes in cognitive and/or neurologic function in the offspring to cretinism (1). Importantly, even mild and moderate degrees of ID may have adverse effects on the neurodevelopment of children (2), and ID is still considered the leading cause of preventable mental impairment worldwide (3). ID is easy to prevent, especially in vulnerable populations such as pregnant and lactating women, through the use of multivitamin supplements that contain sufficient amounts of potassium iodide or iodate. However, the content of multivitamin products is not regulated and among marketed products there may be differences in the iodine content declared on the product label and the measured concentration (4).

In 1922, Switzerland was the first country to fortify household salt with iodine to control endemic goiter and cretinism. In 1952, iodized salt was available across the whole country and, in response to decreasing salt consumption, the iodine amount in household salt was progressively increased from 3.75 mg/kg in 1952 to 25 mg/kg in 2014, resulting in the elimination of ID disorders in Switzerland (5). Swiss policy mandates iodization of salt on a voluntary basis, i.e. both iodized salt and non-iodized salt must be available. Salt samples from households were collected in the framework of multiple national studies between 1999 and 2015 (6–9). The results formally demonstrated that more than 80% of households use iodized salt. However, data on the current use of iodized and non-iodized salt by the Swiss food industry and canteens are limited; they suggest that coverage with iodized salt is incomplete and that many foods are prepared with non-iodized salt (data of Les Salines Suisses SA, the leading salt producer and supplier in the country (10)).

The urinary iodine concentration (UIC) is an excellent biomarker of the dietary iodine intake at the population level. The World Health Organization (WHO) defines sufficient iodine intake in a population as a median UIC ≥100 µg/l in adults and children, and ≥150 µg/l in pregnant and lactating women (3). Periodic national UIC surveys performed in Switzerland in 1999 and 2009 have shown borderline deficient iodine intake in pregnant women (8, 11), as well as in lactating women (11). In a cross-sectional national study performed in 2015-2016, the median UIC in women of reproductive age (n= 353) was 88 μg/L (bootstrapped 95% confidence interval (CI) 72, 103 μg/L), and in pregnant women (n = 363) it was 140 μg/L (bootstrapped 95% CI 124, 159 μg/L) (7). Among women of reproductive age, 92% reported using iodized salt, but only 1.4% used iodine-containing supplements. Among pregnant women, 37% used iodine-containing supplements, 62% did not, and 1.4% did not know. In pregnant women, the median thyroglobulin level (23.8 µg/l), which can be used as a biomarker for iodine status at the population level, was above the concentrations usually found in iodine-sufficient populations; it was elevated in 13% of the pregnant women, thus suggestive of increased thyroid activity. In aggregate, these findings suggest that the iodine intake is only borderline sufficient in pregnant and non-pregnant women in Switzerland (7).

In adults and adolescents, the recommended daily allowance of iodine is 150 µg (3). The Swiss Confederation and the World Health Organization recommend 250 µg of iodine during preconception, pregnancy and lactation (3, 12), the United States National Academy of Medicine (NAM) (formerly the Institute of Medicine (IoM)) recommends a daily iodine intake of 220 μg during pregnancy and 290 μg during lactation (13). The Swiss Society of Endocrinology and Diabetology (SSED) and The American Thyroid Association (ATA) recommend that women who are planning pregnancy, pregnant, or breastfeeding take a prenatal multivitamin containing 150 μg iodine per day to supplement the nutritional iodine intake (13). In these products, the recommended amount of 150 µg iodine is most commonly supplied by adding potassium iodide, which contains 76% iodide. The Swiss Society for Gynecology and Obstetrics does not specifically recommend iodine supplementation but suggests that women use iodized salt during pregnancy.

## Materials and methods

### Iodine content of multivitamin preparations

Using online resources, including the Swiss Compendium for drugs (https://compendium.ch/), we identified adult multivitamin supplements that are commonly available in Switzerland. The products were categorized into 1) products recommended for pregnancy and lactation (prenatal vitamins (PMV), and 2) other widely available multivitamin products for adults. Advice was sought from obstetricians to ensure the list includes the most frequently prescribed supplements for pregnant and lactating women in Switzerland. The contents and the amounts of the ingredients of every product were extracted and tabulated.

## Results

**Table 1** summarizes the iodine content, as well as the amount of selenium, a trace element that is essential for the normal function of selenoenzymes, including the deiodinases.

**Table 1:** Iodine and selenium content of the most frequently used multivitamin products in Switzerland*.

Products recommended for pregnancy and lactation		
	Iodine	Selenium
Recommended Daily AllowanceDuring Pregnancy and Lactation (µg/day)	250	60
Actilife Pronatal	150	–
Andreafol	–	–
Andreavit	200	60
Bonal Folic	–	–
Burgerstein “Pregnancy & Lactation” (2 caps)**	150	55
Centrum prenatal	220	60
Elevit Pronatal	–	–
Elevit Pronatal Complex + DHA	150	60
Ergynatal Nutergia (2 caps)	75	11
FeminaBiane Conception (1 caps + 1 tablet)	150	–
Fol-Ino Women	–	–
Gametix F Femme	150	50
Gestarelle G3	150	–
Gynefam Plus XL	150	–
LadyBiane Maternity	150	–
Natalben Plus (Effik)	250	–
Omnibionta Pronatal+	150	30
Pre Natalben Préconception (Effik)	–	–
Premavid	200	60
Proxeed Women	–	27.5
Pure SS-Box	150	55
Serenatal G Pregnancy	–	–
Solgar Prenatal (2 tablets)	75	12.5
**Widely available multivitamin products**		
	Iodine	Selenium
Recommended Daily AllowanceDuring Pregnancy and Lactation (µg/day)	250	60
Actilife All In One Depot	–	17
Actilife All in One Orange	–	17
Actilife All in One Power Boost	45	20
Actilife Multivit Orange	–	–
A-Z Depot Multivitamines (Doppelherz)	100	30
Berroca	–	–
Burgerstein CELA	150	55
Burgerstein Multivitamin	–	–
Burgerstein TopVital	–	50
Centrum	150	–
Centrum Femmes	150	55
Dextrose Instant Energy Multi-Vitamine	–	–
Dialvit	–	–
Fortevital Tonique	–	17
Pharmaton Vital	–	50
Pharmaton Vital Geriavit	-	-
Supradyn Energy***	75	55
Supradyn Pro Energy Complex***	150	55
Supradyn Vital 50+***	60	28
Vitamin A-Zink Well & Active (Aldi Suisse)	100	25
Vitarnin	–	–

*As of September 2022.

**“Pregnancy and Lactation” - Sold as Grossesse & Allaitement (French) and Schwangerschaft & Stillzeit (German).

***Supradyn products are non exhaustive.

**Figure 1:**
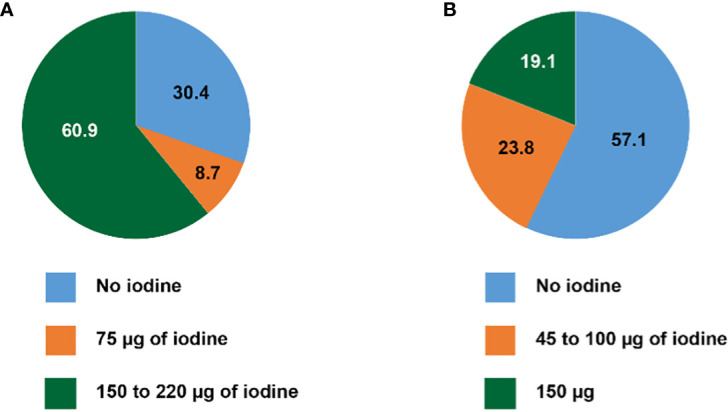
Iodine content of **(A)** multivitamin products recommended for pregnancy and lactation, and **(B)** widely available multivitamin products for adults (in percent).

As illustrated in **Figure 1**, among prenatal multivitamins (PMV), 16/23 (69.6%) contain iodine according to their label. Fourteen out of 23 (60.9%) products contain 150 to 220 µg of iodine, matching or exceeding the recommendations of the SSED and the ATA. Two of 22 (8.7%) contains only 75 µg of iodine, and 7/23 (30.4%) products contain no iodine; surprisingly, the latter group includes the most widely recommended pregnancy supplement.

Among adult multivitamins (AMV), 9/21 (42.9%) contain iodine. Twelve out of 21 (57.1%) contain no iodine, and 5/17 (23.8%) 100 µg of iodine or less (from 45 to 100 µg) (**Figure 1**). Four of 21 (19.1%) products contain 150 µg of iodine, reaching the recommended daily iodine intake for adults, and the supplementation recommended for pregnant and lactating women. Thus, in total, only 18/44 (40.9%) of all multivitamin supplements contain 150 µg of iodine or more.

## Discussion

Despite the important consequences of ID during pregnancy and lactation, only 14/23 (60.9%) of vitamins supplements intended for women who are planning pregnancy, pregnant or lactating, contain at least 150 µg of iodine. Considering the products that are not labeled for specific use in pregnant and lactating women, only 9/21 (42.9%) contain iodine and among the iodine-containing multivitamins, the iodine content is variable with a range from 45 to 150 µg (**Table 1**).

Given that the iodine intake is currently only borderline sufficient in pregnant and non-pregnant women in Switzerland (7), despite the increase in iodine fortification of salt in 2014, the recommendation of the Swiss Society for Gynecology and Obstetrics to use iodized salt during pregnancy, yet not specifically recommending iodine supplementation during pregnancy, should be questioned.

## Conclusions

In conclusion, physicians following pregnant and lactating women should be aware of the variability in iodine content of multivitamin products, and it is recommended to prescribe supplements that contain ≥150 µg of iodine. Manufacturers of prenatal multivitamins are encouraged to review their products and, if the iodine content is insufficient, appropriately enrich their products adhering to established recommendations.

## Data availability statement

The original contributions presented in the study are included in the article. Further inquiries can be directed to the corresponding author.

## Author contributions

Conceptualization, GC, PK, MG; methodology, MG, PK; formal analysis, MG, PK, GC; writing—original draft preparation, MG, PK; writing—review and editing, PK, MG, GC. All authors contributed to the article and approved the submitted version.
