# Erratum: Iodine content of frequently used prenatal and adult multivitamins in Switzerland

**DOI:** 10.3389/fendo.2023.1172672

**Published:** 2023-03-08

**Authors:** Frontiers Production Office

**Affiliations:** Frontiers Media SA, Lausanne, Switzerland

**Keywords:** iodine, iodine deficiency, pregnancy, thyroid function, multivitamins

An omission to the funding section of the original article was made in error. The following sentence has been added: “Open access funding was provided by the University of Lausanne”.

The original version of this article has been updated.

